# The Impact of Hypromellose on Pharmaceutical Properties of Alginate Microparticles as Novel Drug Carriers for Posaconazole

**DOI:** 10.3390/ijms241310793

**Published:** 2023-06-28

**Authors:** Katarzyna Kruk, Marta Szekalska, Anna Basa, Katarzyna Winnicka

**Affiliations:** 1 Department of Pharmaceutical Technology, Medical University of Białystok, Mickiewicza 2C, 15-222 Białystok, Poland; katarzyna.kruk@umb.edu.pl (K.K.); marta.szekalska@umb.edu.pl (M.S.); 2 Institute of Chemistry, University of Białystok, Ciołkowskiego 1K, 15-245 Białystok, Poland; abasa@uwb.edu.pl

**Keywords:** alginate, hypromellose, posaconazole, microparticles, mucoadhesive formulations

## Abstract

Fungal infections are a group of diseases which are challenging to treat because of drug-resistant fungi species, drug toxicity, and often severe patient conditions. Hence, research into new treatments, including new therapeutic substances and novel drug delivery systems, is being performed. Mucoadhesive dosage forms are beneficial to improving drug bioavailability by prolonging the residence time at the site of application. Sodium alginate is a natural polymer with favorable mucoadhesive and gelling properties, although its precipitation in acidic pH significantly disrupts the process of drug release in gastric conditions. Hypromellose is a hydrophilic, semi-synthetic cellulose derivative with mucoadhesive properties, which is widely used as a control release agent in pharmaceutical technology. The aim of this study was to evaluate the impact of hypromellose on alginate microparticles with posaconazole, designed to modify drug release and to improve their mucoadhesive properties for both oral or vaginal application.

## 1. Introduction

Fungal diseases affect patients in both their milder varieties, such as superficial or vaginal, and more severe invasive infections, which are more difficult to treat and might result in high mortality [1]. In general, fungal infections are not easy to treat because there are few groups of antifungal agents available, although research in this field is constantly being performed. Another reason is drug-resistant fungi strains. In recent decades, more infections caused by opportunistic fungi have occurred in patients with reduced immunity [2]. Moreover, the human vulnerability to fungal infections has increased with developments in medicine and the utilization of immunosuppressive therapies [3]. On the other hand, vaginal candidiasis is also a very common fungal infection affecting female reproductive tracts, mostly caused by *Candida* species. Although *C. albicans* naturally exists in vaginal microbiota, it may be difficult to treat fungal growth in certain risk conditions, such as immune deficiency and as a result of antibiotic therapy. The effectiveness of vaginal drug forms is often not sufficient because of short drug residence time at the site of application. Oral antifungal drugs are also utilized in mycosis treatment; however, they are often characterized by low and volatile bioavailability. Applying mucoadhesive microparticles as drug carriers for antifungals might improve the effectiveness of the therapy.

Mucoadhesive drug forms are gaining increasing attention since they provide an extension in residence time at the site of application, consequently prolonging the release of the drug in contact with the mucosa and reducing the frequency of dosing [4,5]. The drug bioavailability is improved, as the formulation is able to release the drug over a sustained period of time [4,6]. Moreover, mucoadhesive drug forms might also act as adjuvants by coating and moisturizing the mucosa [7]. In mucoadhesive drug form preparation, a number of hydrophilic polymers, such as alginates or cellulose derivatives, are employed. Bonding between the polymer and mucosal glycoprotein-mucin might occur in various interactions: ionic, covalent, hydrogen, or van der Waals forces [8]. Mucoadhesive drug forms include tablets, films, solid inserts, micro- and nanoparticles, in situ gelling systems, hydrogels, sprays, and patches.

Sodium alginate (ALG) is a natural polymer with an anionic character and favorable mucoadhesive properties. It is characterized by a linear structure, consisting of mannuronic and guluronic acid residues arranged alternately and linked by β-1,4-glycosidic bonds. ALG is most frequently obtained by *Phaeophyta* algae extraction and less rarely by bacterial synthesis. The numerous qualities of this polymer include high water solubility, beneficial mucoadhesive properties, non-toxicity, biocompatibility, inert character, ease of gelation, properties favorable to spray drying, good availability, and relatively low cost. On the other hand, pure ALG formulations do not provide high mechanical strength, and precipitate at low pH (<3), which is a critical obstacle limiting drug release from the ALG matrix. In order to improve posaconazole’s release profile in an acidic medium, ALG could be blended with a hydrophilic polymer, such as hypromellose [9,10,11].

Hydroxypropyl methylcellulose (HPMC) is one of the most commonly utilized hydrophilic cellulose derivatives in the pharmaceutical industry [12]. HPMC is a hydroxypropylmethyl ether of cellulose accessible in several varieties which differ in viscosity. It is an electronegative polymer, and is characterized by good solubility in cold water with the formation of a colloidal viscous solution [13]. In addition, it is classified as generally recognized as safe (GRAS) by the FDA and EMA [14,15]. Moreover, the polymer has the ability to swell and form a gel structure with mucoadhesive properties [16]. Due to the lack of electrostatic charge, HPMC exhibits a reduced risk of drug interactions and is independent of conditions in the gastrointestinal tract, and therefore can provide repeatable results in release assays. Due to its mucoadhesive properties and gelling ability, it is being investigated from the perspective of applications in oromucosal drug forms, as it rapidly gels upon contact with body fluids with the formation of a gel structure strong enough to protect the matrix from rapid erosion and delay drug release [12]. Microparticles with HPMC have been designed for nimesulide, cimetidine, and vancomycin [17,18,19].

Posaconazole (POS) is an antifungal drug belonging to the azole group, similar in structure and properties to itraconazole [20]. The mechanism of its action involves the inhibition of ergosterol synthesis in fungal cell walls. Among azole derivatives, POS is remarkable for its broad-spectrum antifungal activity against *Aspergillus* spp., *Coccoidioides immitis*, *Fonsacea pedrosoi*, *Fusarium* spp. POS has been approved by the FDA for oral use as 100 mg extended-release tablets and 40 mg/mL oral suspension, and for intravenous administration as 300 mg/16 mL solutions. Indications for the POS application include primarily the prevention of invasive fungal infections with *Aspergillus* spp. and *Candida* spp. [21]. POS is classified as a class II in Biopharmaceutics Classification System (BCS) [22], which means it is well absorbed across biological membranes, but poorly soluble in water, and shows a low, fluctuating bioavailability after oral administration [23,24]. The aim of this paper was to design a multicompartment drug delivery system for POS with favorable release profile and mucoadhesive properties in two environments simulating gastric and vaginal conditions. For this objective, ALG and ALG/HPMC microparticles were formulated by spray drying and the influence of HPMC on the properties of obtained formulations was evaluated. Swelling index, mucoadhesive properties, and release profiles were investigated in simulated gastric fluid (SGF, 0.1 M HCl) and simulated vaginal fluid (SVF). In addition, microparticles were examined using scanning electron microscopy (SEM) and differential scanning calorimetry (DSC). An important aspect was also the in vitro testing of antifungal activity of designed particles using *C. albicans*, *C. krusei*, and *C. parapsilosis* strains.

## 2. Results and Discussion

With the advancement of clinical mycology, there are more and more treatment options available for fungal infections, which are still a clinically significant problem. The development of pharmacology is resulting in the discovery of modern antifungal drugs with different mechanisms of action from conventional drugs, as well as diagnostic tools which improve the entire process of therapy. On the other hand, new strains of fungi have appeared (*Candida auris*, *Lomentospora prolificans*), and the well-known ones have acquired resistance to the antifungal drugs applied (such as *Aspergillus* becoming resistant to azoles). Thus, it is still an open issue and new therapies are being investigated. As a result of particle size reduction, developed contact area with mucosa, and prolonged retention time at the application site, multicompartment drug forms with mucoadhesive properties, such as microparticles, might enhance antifungal drug bioavailability and improve the effectiveness of the therapy [25].

### 2.1. Microparticle Characterization

**Figure 1:**
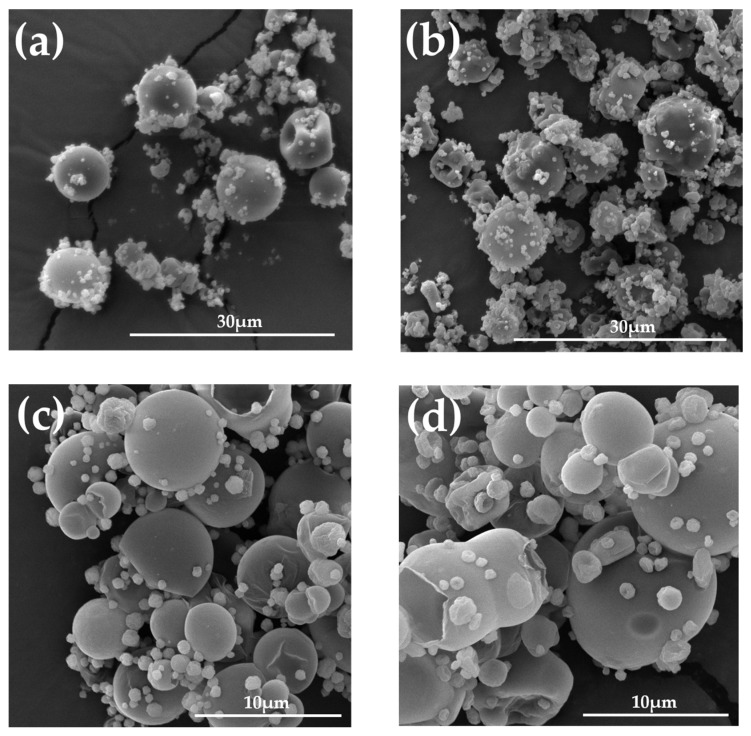
Representative SEM images of microparticle formulations A1 (**a**), F6 (**b**) under magnification 5000×, H1 (**c**), and H5 (**d**) under magnification 10,000×.

The morphology of microparticles was evaluated using SEM technology. ALG placebo particles were mostly characterized by a spherical shape and a homogeneous smooth surface with rare concave inclusions (Figure 1a). In contrast, slightly more irregularly shaped particles were observed among ALG formulations with POS (Figure 1b). ALG/HPMC placebo microparticles possessed mostly a homogeneous spherical shape; however, SEM images reveal more irregularly shaped particles, occasionally with a wrinkled surface (Figure 1c). The images of ALG/HPMC/POS particles show mostly spherical particles with smooth homogeneous surfaces, varying in size with characteristic minor protuberances, whereas fewer particles with indentations may be observed (Figure 1d). In contrast to ALG POS-containing microparticles, among ALG/HPMC drug-containing formulations, several ripped particles were observed (Figure 1d).

**Figure 2:**
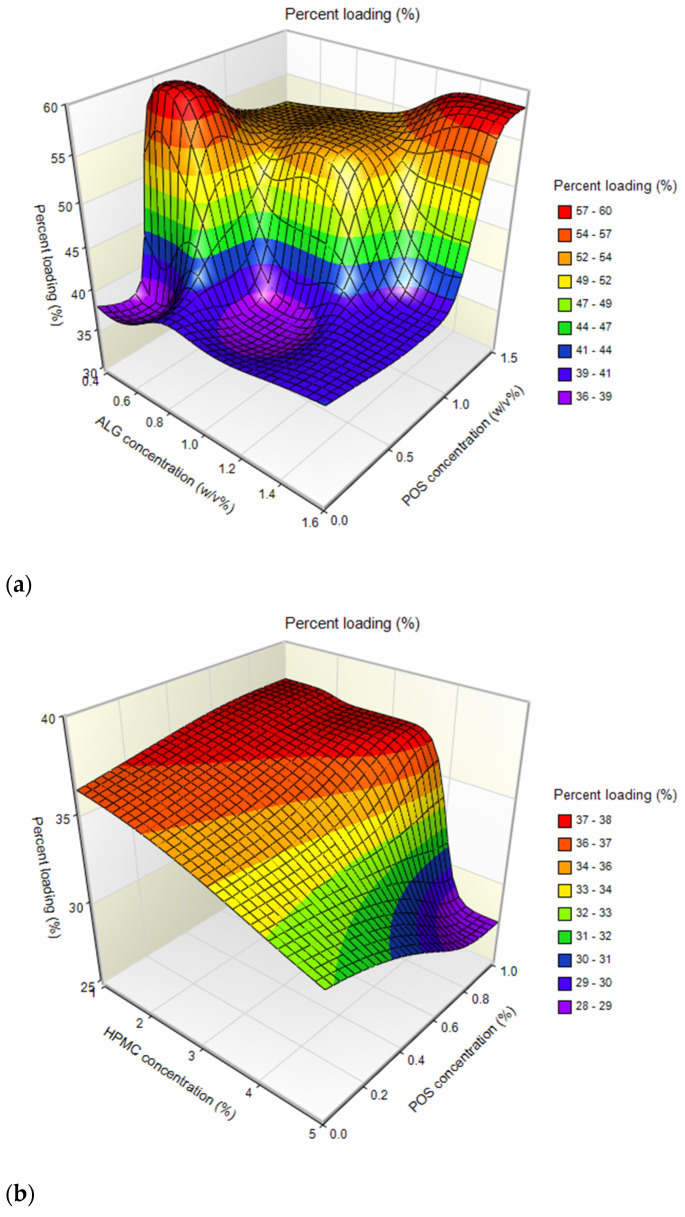
Three-dimensional response surface plots: (**a**) percent loading vs. ALG and POS concentrations and (**b**) percent loading vs. HPMC and POS concentrations.

**Figure 3:**
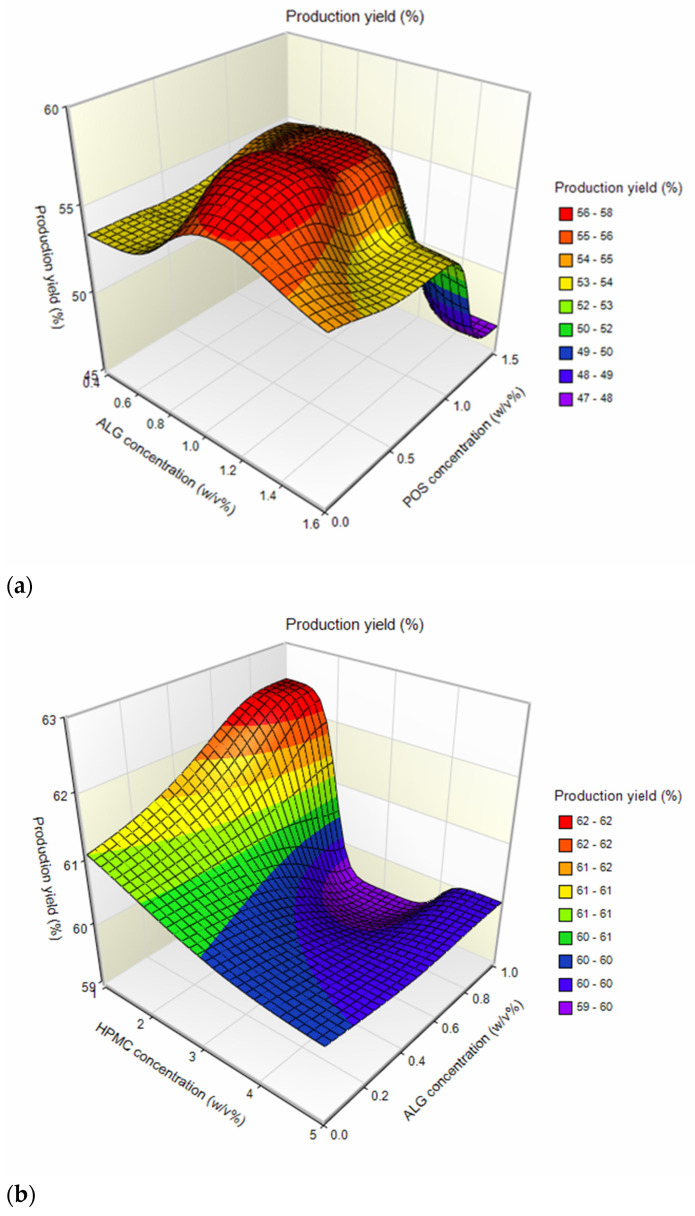
Three-dimensional response surface plots: (**a**) production yield vs. ALG and POS concentrations and (**b**) production yield vs. ALG and HPMC concentrations.

Among ALG placebo formulations, particle size ranged from 10.03 ± 3.84 µm (A1) to 13.42 ± 5.50 µm (A3) (Table 1). A slight increase in the medium particle size was observed with the increase in ALG concentration. This might be related to the fact that the viscosity of the solutions is one of the parameters affecting the spray-drying process. During the spray drying of higher-viscosity solutions, larger droplets are formed and consequently larger particles are obtained [26]. Formulations containing ALG and POS in a 1:1 ratio were characterized by slightly larger average particle sizes (ranging from 13.19 ± 4.01 µm for F6 to 15.27 ± 5.76 µm for F4) than formulations with a component ratio of 1:0.5 (ranging between 11.70 ± 4.81 µm for F5, and 12.81 ± 5.86 µm for F3). The average size of composed ALG/HPMC particles ranged from 10.53 ± 3.22 µm (H1) to 15.25 ± 5.12 µm (H3); however, the trend of gently increasing particle size with increasing polymer concentration was maintained. A similar trend was noted by Szekalska M. et al. [26], where the fucoidan/gelatin particle size increased with an increase in gelatin content. This trend was equally pronounced in POS-containing formulations, with particle sizes increasing with increasing HPMC concentration in the formulation, from 12.23 ± 4.13 µm (H4) to 15.64 ± 4.48 µm (H6). In order to understand the effect of variable parameters, ALG, HPMC, and POS concentrations on the microsphere properties, multivariate analysis based on the three-dimensional (3D) response surface plots were prepared. This method of data analysis is a useful tool for exploring desirable response values and varying terms [27,28]. Higher drug loading values were reported for formulations of ALG:POS in a 1:1 ratio, compared to a 1:0.5 ratio (values about 1.5 times higher) (Figure 2a, Table 1). Similar results were observed by Yang M. et al. [29], where higher drug loading values were obtained for higher amounts of POS in formulations. Drug loading of mixed ALG/HPMC formulations was decreased compared to the pure ALG formulations. In addition, a decrease in drug loading was evident as the concentration of HPMC increased (Figure 2b). Encapsulation efficiency values (from 112.38 ± 4.22% (F3) to 119.67 ± 3.41% (F5) were insignificantly higher (*p* > 0.05) for the ALG:POS formulations at a 1:0.5 ratio than for the ALG:POS 1:1 formulations (from 108.59 ± 5.18% (F3) to 116.78 ± 4.48% (F6) (Table 1). Values of encapsulation efficiency are typically higher for microparticles obtained via spray drying compared to other methods [30]. Values of this parameter > 100% observed in the study might be caused by the fractional loss of polymer mass during the spray-drying process. As a result, the drug content measured in the study was higher than theoretical [31]. The moisture content of the microparticles was at a relatively low level, and fluctuated from 7.54 ± 0.32% for H1 formulation to 14.23 ± 1.53% for A3. This is an important parameter, especially with regard to the stability of the formulation during storage [32]. The highest production yields were obtained for ALG placebo formulations; at over 60% (from 63.49 ± 2.77% (A2) to 64.47 ± 6.51% (A3)), lower process yields were obtained for ALG POS-containing formulations (Figure 3a, Table 1). A similar correlation was observed for encapsulation efficiency values—lower yields were observed for formulations containing ALG:POS in a 1:1 ratio compared to a 1:0.5 ratio. On the basis of the three-dimensional response surface plot, it was shown that the drying yields of ALG/HPMC formulations decreased compared to ALG formulations (Figure 3b), which is related to the higher viscosity of the composed solutions. Presumably, it resulted in incompletely dried larger particles settling on the chamber walls [32]. However, a significant (*p* < 0.05) increase in yield of POS-containing formulations was observed (from 59.43 ± 1.90% (H5) to 62.46 ± 4.56% (H6)) referring to placebo formulations (from 51.61 ± 4.69% (H2) to 54.06 ± 5.77% (H1)). Compared to other formulations containing POS, such as inert spheres coated with solid dispersion containing POS, microparticles obtained by spray drying were characterized by smaller particle size and higher encapsulation efficiency, which are factors improving drug bioavailability [33].

### 2.2. Swelling and Mucoadhesion

The ability to swell is crucial in terms of the phenomenon of mucoadhesion and the drug release process. Mucoadhesion is defined as the ability of synthetic materials to adhere to the surface of mucous membranes. Polymer contact with moisture is necessary to form bonds with mucin from mucosa and leads to polymer swelling and renders its structure more flexible; as a result, it facilitates the penetration of the mucosa [34].

**Figure 4:**
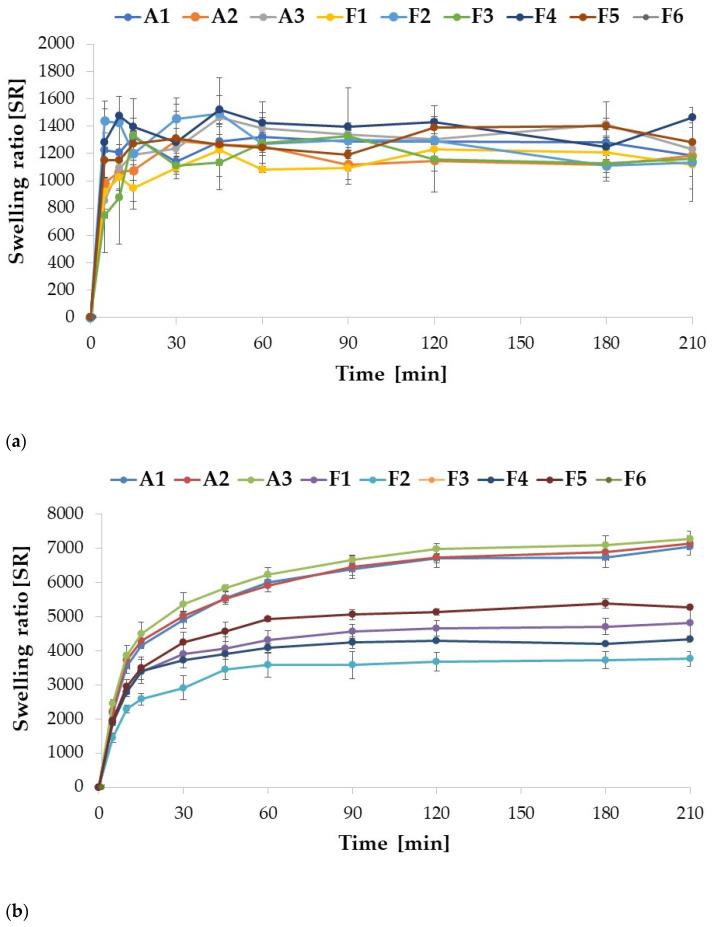
Swelling ratio (SR) of ALG (A1 to F6) formulations assessed in gastric (**a**) and vaginal (**b**) conditions (0.1 M HCl and SVF, respectively) (mean ± SD, *n* = 3).

The swelling assay was conducted in two environments, 0.1 M HCl (pH = 1.2) and SVF (pH = 4.2), to mimic gastric and vaginal conditions, respectively. The ALG placebo formulations A1 and A2 showed a very similar swelling profile, although A1 was characterized by slightly higher (*p* > 0.05) swelling index values at each measurement point (except for the point after 30 min). Among the placebo formulations, the highest swelling rate was recorded for the A3 formulation. Drug-containing formulations swelled more with increased POS content. Similarly to the placebo formulations, swelling enhanced with increasing ALG concentration in POS-containing formulations (Figure 4a). In the 0.1 M HCl medium, the drug incorporation into the formulations improved the swelling index of formulations F4 and F6. A swelling peak was observed for each formulation, after which the swelling index values did not change significantly (*p* > 0.05). When pH decreased below ALG pKa values (3.38–3.65) [35], ALG underwent protonation with formation of alginic acid, which is poorly soluble in water [36]. A compacted shell was formed around the microparticles, which hindered water influx and further swelling. The swelling profile of ALG microparticles in SVF appeared differently, since there were no peaks in values reported for any of the formulations tested. Swelling rates increased consistently through the time (Figure 4b). The placebo formulations exhibited significantly (*p* < 0.05) higher (about 1.5 times) swelling compared to the POS-containing formulations. Similarly, when tested in 0.1 M HCl, swelling increased with increasing ALG concentration in the placebo formulation. On the other hand, the opposite trend was noticed in the POS-containing formulations; as the drug content increased, the values of the swelling factor were decreased. This is probably related to the higher solubility of ALG in the SVF environment (pH = 4.2). Among ALG/POS formulations, the highest swelling ratio values were registered for F3 and F5 (around 5000%), while the lowest for F2 (3765%).

**Figure 5:**
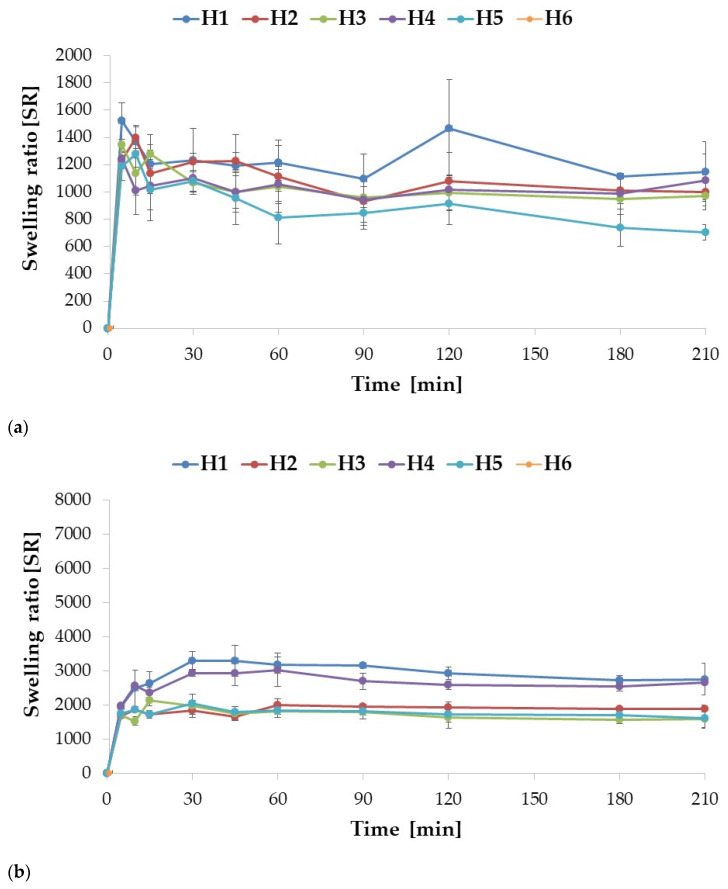
Swelling ratio (SR) of ALG/HPMC formulations (H1 to H6) assessed in gastric (**a**) and vaginal (**b**) conditions (0.1 M HCl and SVF, respectively) (mean ± SD, *n* = 3).

Enrichment of the formulation with HPMC affected the swelling profiles in both environments (Figure 5a,b). In 0.1 M HCl, dissolution of composite ALG/HPMC particles was observed, in contrast to ALG particles, which precipitated in this environment, the more pronounced the higher the HPMC content of the formulation. The formulations with the lowest HPMC content, H1 and H4, did not dissolve completely, and were characterized by the highest swelling ratios after 210 min of the assay (1148% and 1087%, respectively). Moreover, an increase in HPMC concentration resulted in a decrease in the swelling. After reaching the peak (5 min for H1 and H3, and 10 min for H2), the swelling rates consistently decreased in time, with few fluctuations, which could be related to incomplete draining of the basket before weighting. ALG is a polymer which is characterized by pH-dependent swelling properties. At a low pH, carboxylate groups are partially unionized, which results in polymer shrinkage and decreased swelling ability at pH 1.2. The addition of HPMC, which interpenetrates ALG within the microparticles structure, provides improved swelling capacity [37]. HPMC in contact with a moisture environment is characterized by rapid water uptake. In the next step, the polymer is hydrated and the chains gradually unravel. The hydrogen bonds of the polymer are broken, creating available areas for further water binding. As a result of this process, swelling occurs. During prolonged contact with the medium, the creation of gel is observed, which in turn leads to a reduction in water absorption and, as a consequence, to a decrease of swelling properties [14].

The POS-containing formulations were characterized by lower swelling rates compared to placebo; however, no clear inference regarding the effect of HPMC concentration on swelling may be derived from the results obtained. The highest swelling rate values were observed for formulation H4, while the lowest were observed for H5, and this tendency persisted during the entire experiment. The highest swelling values were observed after 10 min (H5 and H6) and 30 min (H4), indicating a slight prolongation of the swelling process after the drug was incorporated into the formulation. These changes may be attributed to swelling and dissolution processes occurring simultaneously. Before the peak was reached, swelling dominated, while after the peak, dissolution began to predominate, and therefore swelling rates began to decrease gradually. In the SVF, a similar tendency was observed; swelling decreased with increasing HPMC concentration in both placebo and POS formulations, comparable to the work of Hussain A. et al. [38], where increased HPMC content in buccal tablets with flurbiprofen and lidocaine resulted in poorer swelling. Abbasi S. et al. [39] also observed a decrease in swelling index after blending ALG with HPMC in buccal tablets with captopril. In contrast to pure ALG formulations, where there was no apparent peak, swelling peaks were observed for each ALG/HPMC formulation, similarly to the results of a swelling study of ALG/HPMC films with abacavir by Ghosal K. et al. [36]. The POS-containing ALG/HPMC combined formulations demonstrated slightly lower swelling compared to the placebo formulations, as did the test in HCl, presumably related to the percentage reduction of polymer in the formulations. The observed swelling values for the combined formulations were lower compared to the pure ALG formulations. Hence, a significant effect of HPMC can be observed, which, by improving and accelerating the solubility of the complex, enhances the swelling process.

**Figure 6:**
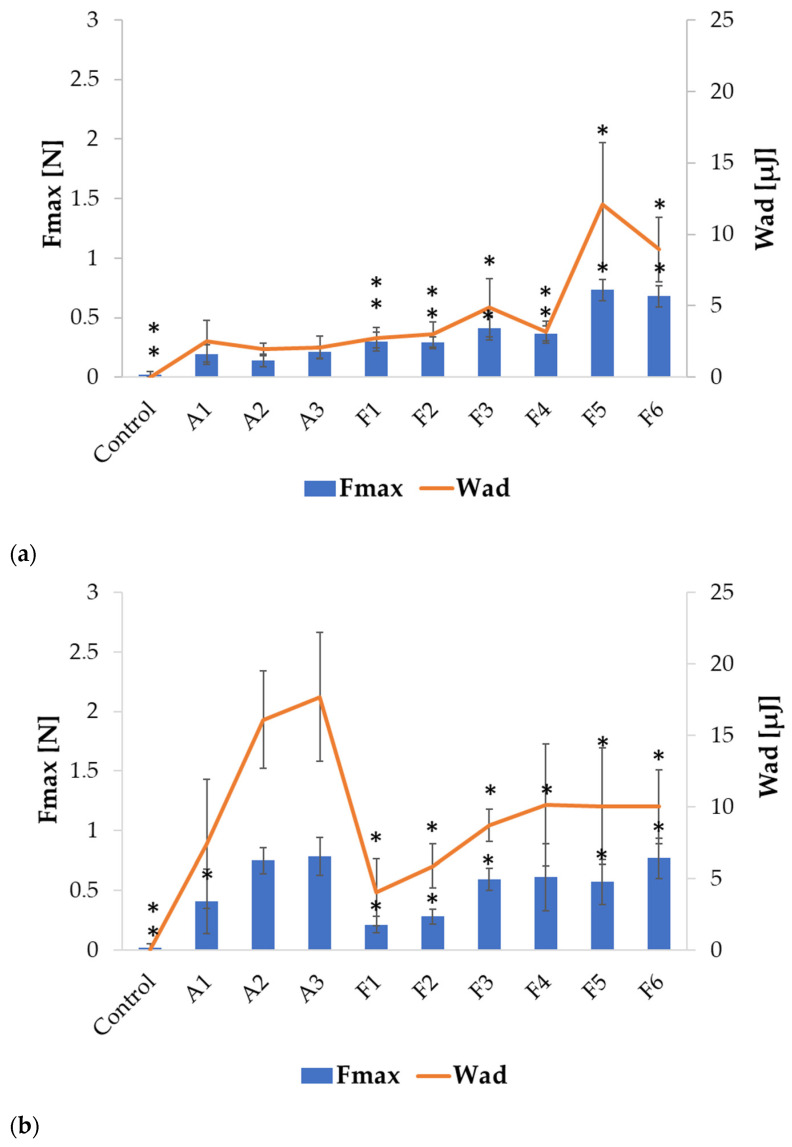
Mucoadhesive properties of ALG formulations (A1 to F6) assessed in gastric (**a**) and vaginal (**b**) conditions (0.1 M HCl and SVF, respectively), (mean ± SD, *n* = 6), * significant differences (*p* < 0.05) compared to formulation A2.

The mucoadhesive properties of the formulated microparticles were evaluated by examining detachment force (F_max_) (N) and work of adhesion (W_ad_) (µJ) using porcine gastric and vaginal mucosa as model adhesion layers. The results presented in Figure 6 reveal that ALG microparticles exhibited mucoadhesive properties in both environments, which increased with increasing ALG concentration, both in placebo and POS-containing formulations. However, in SVF, higher values of tested parameters were observed, similar to the results reported by Szekalska M. et al. [26]. ALG placebo formulations were characterized by lower F_max_ and W_ad_ values, compared to POS-containing formulations in 0.1 M HCl (Figure 6a). Higher values of these parameters were recorded for formulations with lower POS content compared to formulations with higher POS content. The highest F_max_ value was recorded for formulation F5 (0.73 ± 0.09 N), while the lowest was observed for F2 (0.29 ± 0.05 N). In contrast, the opposite trend was observed in the SVF environment, as higher F_max_ and W_ad_ values were reported for ALG placebo formulations than for drug-containing formulations. In addition, stronger mucoadhesion was observed for formulations with higher drug content. The highest F_max_ value was recorded for formulation F6 (0.77 ± 0.17 N), while the lowest was recorded for F1 (0.21 ± 0.07 N). This might be related to the different solubility of ALG in the tested environments. In HCl, the solubility of ALG and swelling ratio were lower, and therefore the mucoadhesive properties of the placebo formulation were lower. In SVF, the solubility and the swelling index of ALG increased, and as a result the mucoadhesive properties were improved (Figure 6b).

**Figure 7:**
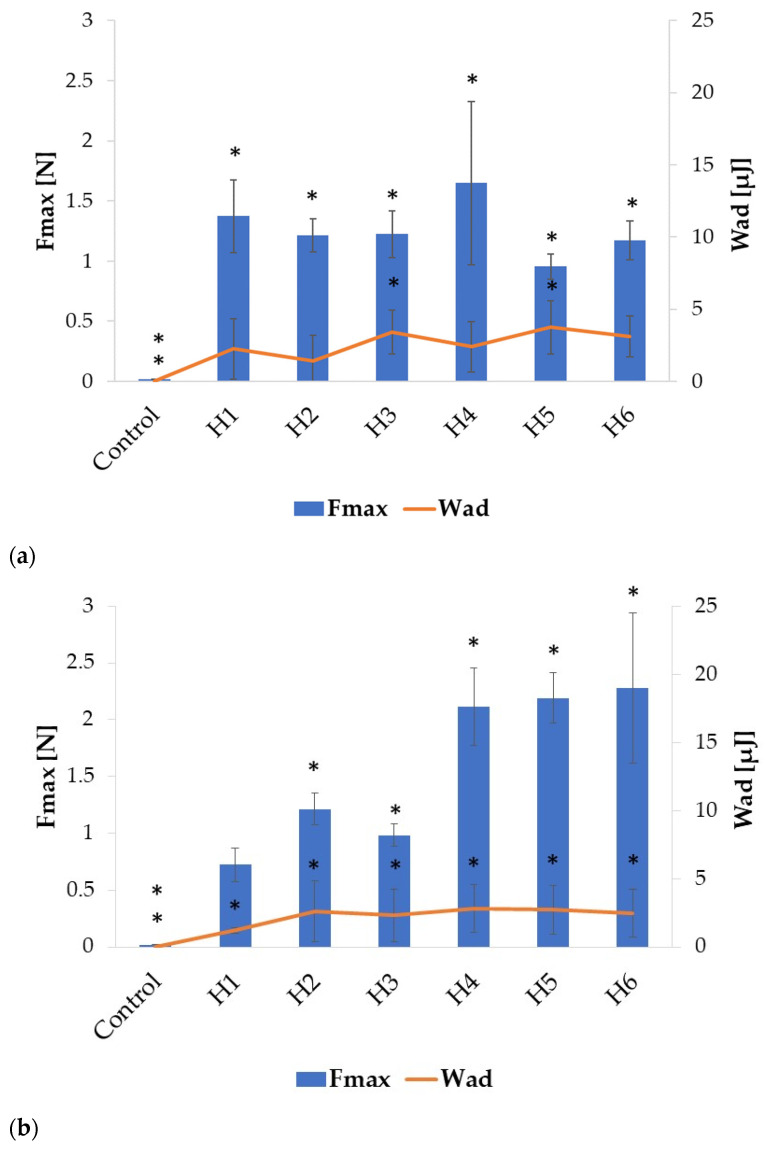
Mucoadhesive properties of ALG/HPMC formulations (H1 to H6) in gastric (**a**) and vaginal (**b**) conditions (mean ± SD, *n* = 6), * significant differences (*p* < 0.05) compared to formulation A2.

The introduction of HPMC into the formulation altered the mucoadhesive properties of the fabricated microparticles, as is presented in Figure 7. An increase in the detachment force and work of adhesion was observed in both tested environments. HPMC belongs to the group of non-ionic polymers, whose mucoadhesive properties are not pH-dependent, but depend on the bond strength of both layers and the entanglement of polymer and mucin chains. Although usually non-ionic polymers do not provide as strong adhesion as ionic polymers, in a low pH environment, higher HPMC swelling properties than ALG might facilitate contact with the adhesive layer, resulting in improved adhesion parameters made by ALG/HPMC microparticles [12].

Among all compounded formulations in the HCl medium, the highest F_max_ values were recorded for those with the lowest HPMC content, both among placebo and POS-containing formulations: H1 (1.37 ± 0.24 N) and H4 (1.65 ± 0.64 N) (Figure 7a). As the concentration of HPMC increased, the mucoadhesive properties of the placebo and drug-containing microparticles deteriorated slightly, presumably due to the increased viscosity and formation of a compact gel structure on the microparticles’ surface, limiting fluid influx and the swelling process. Similarly to ALG microparticles, mucoadhesion increased after changing the environment to SVF (Figure 7b). The placebo formulations were characterized by significantly lower (*p* < 0.05) values of the tested parameters compared to the drug-containing ones. With increased HPMC content, F_max_ values were also increased, from 2.11 ± 0.32 N for H4, through 2.19 ± 0.2 N for H5, to 2.28 ± 0.62 N for H6, similarly to the results obtained in another study [38]. The obtained results showed that the combination of ALG with HPMC improved the mucoadhesive properties of the designed microparticles. Blending HPMC with ALG in buccal films also resulted in an enhancement in mucoadhesive properties in the study of Okeke O.C. et al. [40].

### 2.3. In Vitro POS Release

The release study is a crucial step in the drug formulation design process, as it enables the evaluation and prediction of the behavior of the formulation in a specific medium. Although the assay does not reflect the in vivo conditions, it allows us to predict the behavior of the formulation. Similarly to most drugs, POS is poorly soluble in water, and therefore the limiting step in the absorption process is the dissolution rate. With the use of in vitro models, based on the evaluation of the dissolution rate of the therapeutic substance, it is possible to predict the absorption of the drug in the gastrointestinal tract. The use of a mucoadhesive drug form, by prolonging contact with the mucosa, might prolong drug release at the site of application and increase its bioavailability.

**Figure 8:**
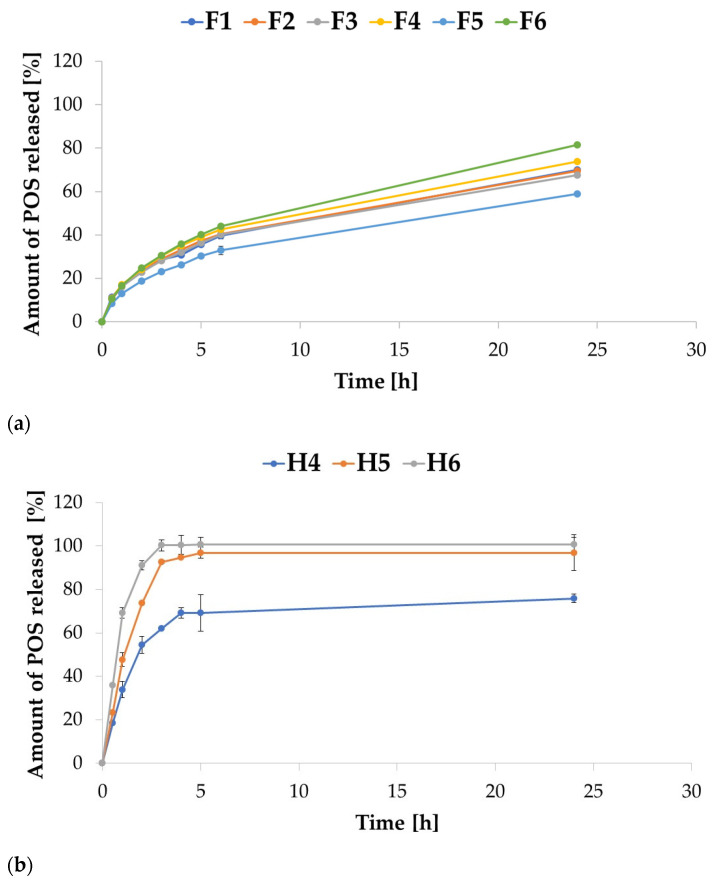
POS release profiles from ALG (**a**) and ALG/HPMC (**b**) microparticles in 0.1 M HCl imitating gastric conditions (mean ± SD, *n* = 3).

The release study for ALG microparticles in 0.1 M HCL imitating gastric conditions showed that ALG:POS microparticles at a ratio of 1:1 (F2, F4, and F6) were characterized by increased POS release compared to 1:0.5 ALG:POS formulations (F1, F3, and F5). Therefore, as the ALG concentration increased, POS release decreased for formulations F1, F3, and F5, containing ALG:POS in a 1:0.5 ratio, while the opposite tendency was displayed for formulations F2, F4, and F6, containing these components in a 1:1 ratio. After 24 h, release ranged from 58.95% for F5 formulation to 81.55% for F6 formulation. The release increased as the ALG concentration increased. Petchsomrit A. et al. [41] reported a similar tendency that increased drug and ALG content in ALG-based composite sponges, resulting in increased drug release. The release profiles from all ALG formulations were comparable, the drug released slowly from the drug form (Figure 8a). After the first hour of the study, the amount of released POS ranged from 8.43% for formulation F5 to 11.44% for F1. After 6 h, the amount of drug released fluctuated around 40% for all formulations, except F5, for which 32.87% was recorded, and the highest amount of POS was released from F6—43.92%. At the final point of the assay, only the F6 formulation achieved a drug release of more than 80–81.55%. For all ALG formulations, the POS release was incomplete. At the end of the study, formulation aggregates were still visible with a coating shell formed by ALG precipitation in the acidic environment, which presumably restricted the diffusion of the drug substance to the release medium. In addition, the formulations with the highest POS release after 24 h (F4-73.76% and F6-81.55%) also exhibited the highest swelling index values. The POS release profile in 0.1 M HCl was affected by HPMC. ALG/HPMC formulations were characterized by faster drug release in 0.1 M HCl medium compared to ALG-only formulations (Figure 8b). After 3 h of running the assay, 100% release from the H6 formulation was observed, at which time 92.78% of the released drug from the H5 formulation was recorded. The highest percentage of released drug for the H4 formulation was observed after 24 h—75.9%. As the concentration of HPMC increased, an increase in the release rate of POS was observed, since the addition of a hydrophilic polymer facilitated fluid influx into the microparticles mass, and improved the solubility of the ALG formulations, and as a consequence accelerated the release process. Yong et al. [42] indicated that HPMC was characterized by weaker drug binding compared to ALG. A complete drug release from the H6 formulation was observed. Incomplete POS release from H4 formulation was observed after 24 h and an undissolved formulation residue was noticed in the test basket, indicating incomplete dissolution of the drug form despite the addition of HPMC.

**Figure 9:**
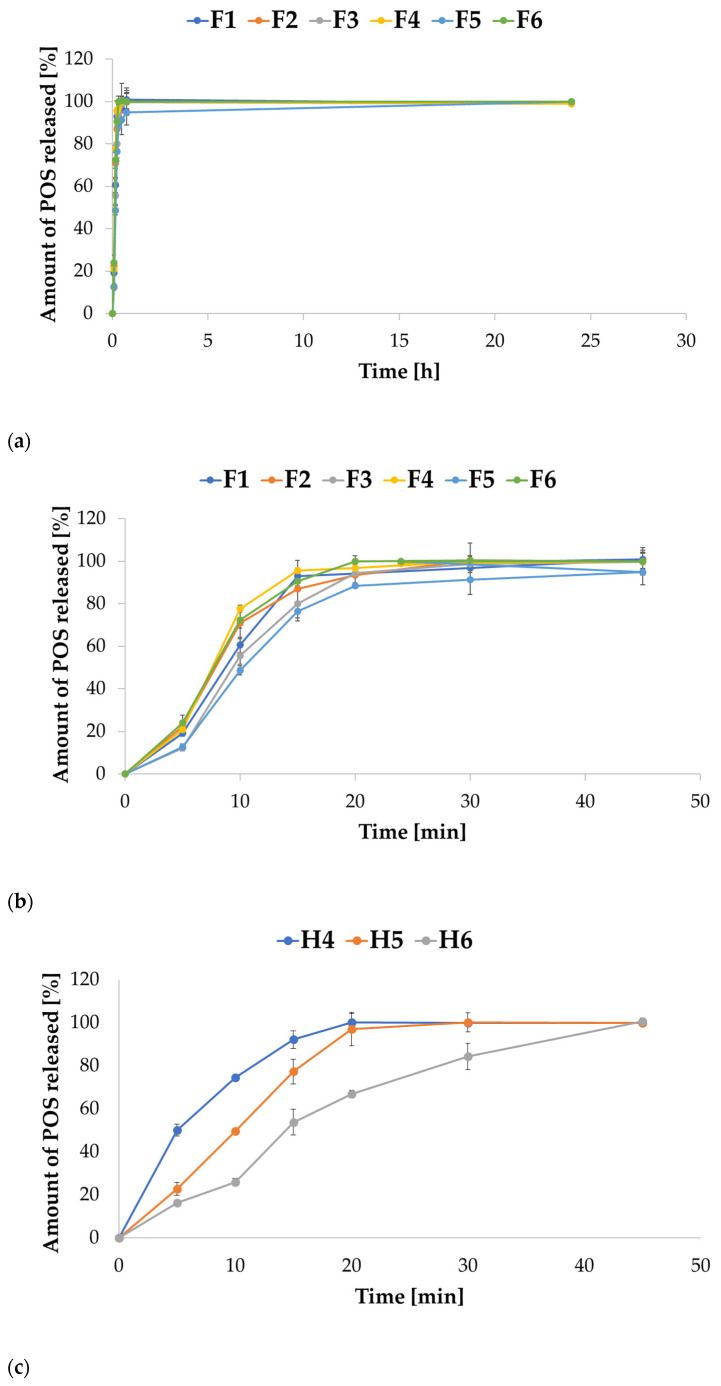
POS release profiles from ALG (**a**,**b**) and ALG/HPMC (**c**) microparticles in SVF imitating vaginal conditions within 24 h (**a**), and within 45 min (**b**,**c**) (mean ± SD, *n* = 3).

The release study was further carried out in SVF, which simulated vaginal conditions (Figure 9). POS release in this environment, from both ALG and combined ALG/HPMC formulations, was significantly faster (*p* < 0.05) than in 0.1 M HCl, which is related to the greater ALG solubility and the stronger swelling of the formulations in the medium. In contrast to the results of the release study conducted in 0.1 M HCl, 100% release of POS from all formulations was observed in SVF. POS release ranged from 12.18% for F4 formulation to 24.02% for F6 formulation after 5 min. The fastest drug release was captured for F6 formulation; 100% of the released drug was recorded after 20 min. Formulations F2 and F4 reached 100% of release after 30 min, while F1 and F3 reached 100% of release after 45 min (Figure 9b). Compounded formulations were characterized by similarly rapid POS release. Complete drug release was observed after 20 min for H4 formulation, after 25 min for H5 formulation, and after 30 min for H6 formulation (Figure 9c). Buccal ALG/HPMC films with cetirizine dihydrochloride obtained by Pamlényi K. et al. [43] also dissolved quickly; almost 100% of drug release in 20 min. The results obtained are in accordance with the results of the swelling study, where the strongest swelling was observed for H4 formulation, while the weakest was observed for the for H6 formulation; the higher the swelling index, the faster the drug release was observed. As the concentration of HPMC in the formulation increased, the drug release rate decreased, andthus more extended drug release was provided.

The results obtained from the release study were applied to modeling using various mathematical equations: zero-order and first-order kinetics, Higuchi, Hixson–Crowell, and Korsmeyer–Peppas models to evaluate the release mechanism of POS (Table 2). The modeling demonstrated that the release mechanism was dependent on the medium employed and the microparticle composition. Regarding ALG microparticles, the results of the release assay in both 0.1 M HCl and SVF environments showed concentration-dependent POS release according to the first-order kinetics. In addition, data obtained from the Higuchi model demonstrated high R^2^ values, suggesting drug release in accordance with Fickian diffusion. Moreover, the high R^2^ values obtained in the Hixson model indicated that POS release might be related to diffusion and erosion of microparticles. The low values of diffusion exponent (*n*) < 0.43 in the Korsmeyer–Peppas model in SVF confirmed that POS release occurred by Fickian diffusion. The values obtained during the analysis of drug release from the compounded microparticles indicated the dependence of the release kinetics on the medium employed. The POS release in 0.1 M HCl occurred according to the first-order kinetics, and thus was dependent on the amount of drug remaining in the drug form. The diffusion exponent (*n*) obtained in the Korsmeyer–Peppas model in 0.1 M HCl medium for H4 and H5 formulations indicated POS release by diffusion, while for H6 formulation, the value of *n* = 0.57 suggested a non-Fickian release, since 0.43 < *n* < 0.85 [44]. Changing the environment from 0.1 M HCl to SVF altered the POS release kinetics from the first-order to the zero-order; hence, the drug release was concentration-independent. The high R^2^ values obtained in the Higuchi model suggested Fickian diffusion, and in the Hixson model, mainly for formulations H5 and H6, indicated that release might occur by diffusion combined with microparticle erosion, similarly to the results obtained by Abassi S. et al. [39].

### 2.4. DSC Analysis

**Figure 10:**
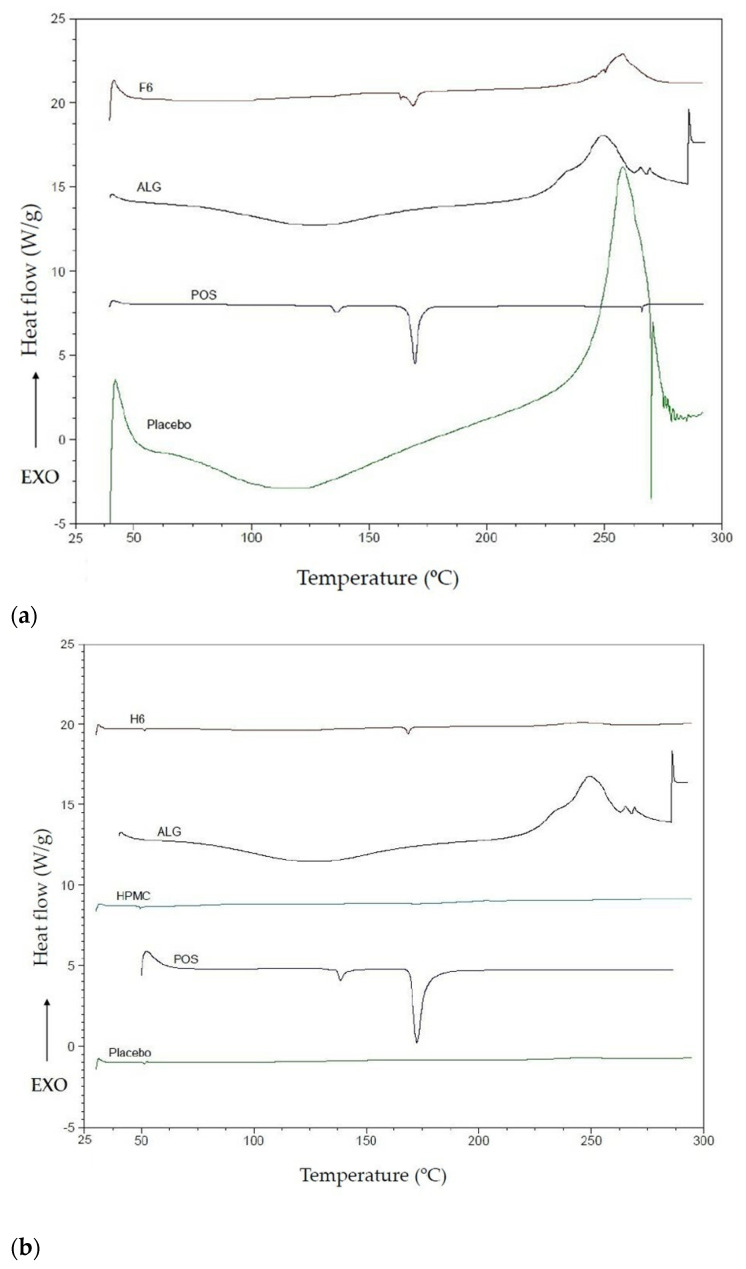
DSC thermograms of ALG, POS, A3, and F6 formulations (**a**); and ALG, HPMC, H3, and H6 formulations (**b**).

Differential scanning calorimetry (DSC) is a method for assessing the thermal properties of drug formulations. Understanding and evaluating phase transitions, analyzing properties such as melting points, is crucial to comprehending the relationship between the polymeric composition of a drug form and its microstructure and stability [45,46]. DSC analysis was performed for pure ALG, HPMC, POS, placebo and drug-containing formulations (Figure 10). Optimal formulations of ALG and ALG/HPMC POS-containing formulations were selected for the study. In the ALG thermogram, a broad endothermic peak was observed in the range of 50–150 °C. It is likely that its occurrence is related to the water evaporation phenomenon during the temperature rise. Moreover, a sharp exothermic peak was registered in the ALG thermogram at 248 °C, which is presumably associated with the degradation of the polymer structure. The explanation for the appearance of this peak proposed by Dudek et al. [47] pointed to the fact of ALG chain decomposition during the dehydration process, and the depolymerization and destruction of saccharide rings due to the partial decarboxylation of ALG carboxyl groups. On the other hand, two sharp peaks were observed in the POS thermogram at 138.7 °C, indicating the loss of crystalline water, and at 172.7 °C, related to the melting point of POS. In the thermogram of the ALG placebo formulation, a characteristic broad endothermic peak was observed between from 50–150 °C and a shift of the peak from 248 °C to 258 °C. In the thermogram of the F6 formulation, two weaker peaks from POS were observed: one shifted from 138.7 °C to 161 °C and the other without shifts at 172.7 °C, and a peak shift from 248 °C to 258 °C originating from degradation of ALG structure. Glass transition temperature of HPMC ranges from 170 °C to 180 °C, and the degradation temperature fluctuates between 200 °C and 250 °C, depending on the HPMC substitution [12]. In the HPMC thermogram, no peaks indicating melting point were noted, suggesting an amorphous state, similarly to other works [48,49]. The DSC curve for the ALG/HPMC placebo formulation showed no change. In the thermogram of the H6 formulation, a sharp shifted peak at 169 °C characteristic of the melting point of POS was registered. A peak originating from the loss of crystalline water from POS was not observed. Peaks at temperature values characteristic of ALG at water evaporation or polymer molecule degradation were not observed in the curves of either the ALG/HPMC placebo formulation or the H6 formulation.

### 2.5. Antifungal Activity

**Figure 11:**
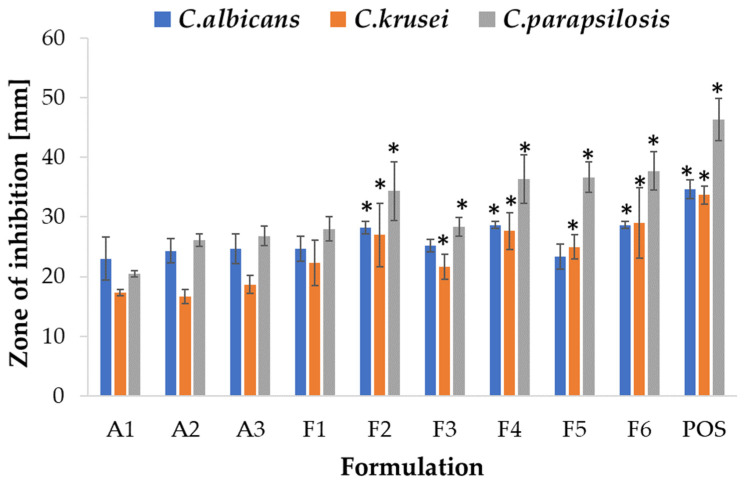
Antifungal activity of ALG placebo microparticles (A1–A3) and drug-containing formulations (F1–F6) against *C. albicans*, *C. krusei*, and *C. parapsilosis* with POS in dimethylsulphoxide (DMSO) as a control (mean ± SD, *n* = 3) * significant differences (*p* < 0.05) compared to formulation A2.

**Figure 12:**
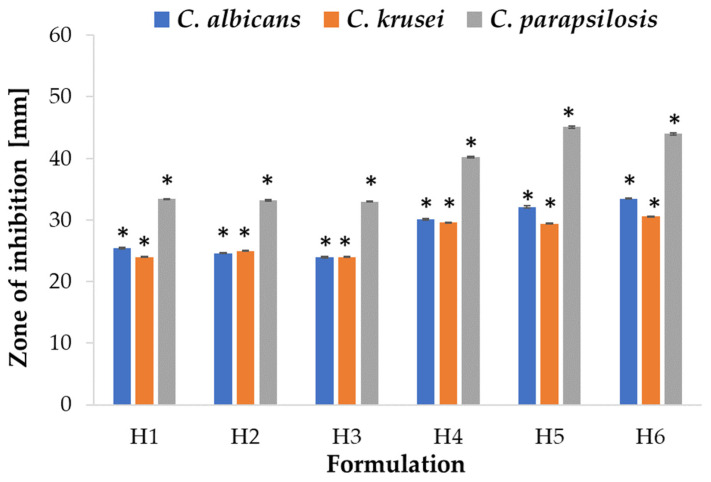
Antifungal activity of ALG/HPMC microparticles (H1–H6) against *C. albicans*, *C. krusei*, and *C. parapsilosis* with POS in DMSO as a control (mean ± SD, *n* = 3) * significant differences (*p* < 0.05) compared to formulation A2.

Due to its favorable properties, such as biocompatibility, biodegradability, mucoadhesiveness, and non-toxicity, ALG is utilized as an antifungal drug carrier; however, there is limited information indicating ALG antifungal activity [50]. The mechanism of ALG antifungal activity might be related with the inhibition of biofilm formation and an enhancement of the sensitivity to antifungal drugs [51]. There are no reports on the antifungal effect of HPMC, and there are only few papers on antifungal drug carriers containing HPMC, such as topical emulsion with amphotericin B [52]. ALG and ALG/HPMC placebo and POS-containing microparticles were tested for antifungal activity utilizing a method standardized by the Clinical and Laboratory Standards Institute—an agar diffusion test employing strains of *Candida* spp. [53]. The study revealed that the most sensitive strain to the tested formulations was *C. parapsilosis* in both the ALG and ALG/HPMC formulation tests. Interestingly, the ALG placebo formulations showed antifungal activity against all strains tested (Figure 11). A slight increase (*p* > 0.05) in the inhibition zone was observed with increasing ALG concentration in the placebo formulations. The ALG formulations demonstrated the weakest antifungal activity against *C. krusei* as the smallest zones of growth inhibition were recorded. ALG POS-containing formulations at a ALG:POS ratio of 1:1 displayed larger inhibition zones and therefore stronger antifungal activity against *C. albicans* compared to formulations of 1:0.5 ratio. The opposite trend was observed for *C. krusei* and *C. parapsilosis*. The introduction of HPMC resulted in a slight increase in zones of inhibition against all tested strains (Figure 12). The placebo ALG/HPMC-compounded microparticles exhibited similar antifungal activity against *C. albicans* and increased activity against *C. krusei*, and *C. parapsilosis*, compared to the antifungal activity of pure ALG placebo formulations, which may be related to the increased swelling of the compounded formulations and hence facilitated fluid influx into the microparticles with improved POS release.

## 3. Materials and Methods

### 3.1. Materials

ALG (source of acquisition: *Macrocystis pyrifera*) with medium viscosity (282 mPa·s for 1% solution at 25 °C, 61% mannuronic acid (M), and 39% guluronic acid (G), molecular weight 3.5 × 105 Da, and M/G ratio of 1.56) was obtained from Sigma Aldrich. HPMC (Pharmacoat 615, viscosity 15 cP) was received from Shin-Etsu Chemical Co., Ltd. (Tokyo, Japan). POS was attained from Kerui Biotechnology Co. Ltd. (Xi’an, China). Methanol was procured from Merck (Darmstadt, Germany). Water was obtained by distillation using a Milli-Q Reagent Water System (Billerica, MA, USA). Simulated vaginal fluid (SVF, pH = 4.2) was obtained by dissolving in 1 L of water: 0.018 g bovine albumin, 0.16 g glycerol, 0.222 g calcium hydroxide, 0.4 g urea, 1.0 g acetic acid, 1.40 g potassium hydroxide, 2 g lactic acid, 3.51 g natrium chloride, and 5 g glucose [54]. Sabouraud dextrose agar (SDA) and stock cultures of *C. albicans* ATCC^®^ 10231, *C. krusei* ATCC^®^ 6528, and *C. parapsilosis* ATCC^®^ 22019 from the American Type Culture Collection were delivered from Alchem Biomaxima (Lublin, Poland). Cellulose acetate membrane filters (0.45 µm) were purchased from Millipore (Billerica, MA, USA). Nylon membrane filters (0.45 µm) were obtained from Alchem (Toruń, Poland). Porcine gastric and vaginal mucosa were received from a veterinary service (Turośń Kościelna, Poland). Extracts from porcine mucosa were frozen at −20 °C and stored for a maximum of one month prior to testing. This process did not require the confirmation of the Local Ethical Committee for Experiments on Animals. All other reagents utilized in the experiments were of analytical grade.

### 3.2. ALG and ALG/HPMC Solutions Preparation

ALG solutions were prepared at three different concentrations by dissolving an appropriate amount of ALG in water at room temperature using an RZR 2020 mechanical stirrer (Heidolph Instruments, Schabach, Germany). The viscosity of the obtained solutions was measured and optimal concentration was selected for further studies. Subsequent solutions with a constant concentration of ALG and a variable concentration of HPMC were prepared by dissolving appropriate amounts of ALG and Pharmacoat 615 in water at room temperature with the use of a mechanical stirrer. Viscosity measurements were carried out repeatedly in order to select optimal concentrations.

### 3.3. Viscosity Measurements

Measurements were performed using rotational viscometer Haake Viscotester 6 Plus (Thermo Fisher Scientific, Waltham, MA, USA) at room temperature (22 ± 2 °C) with speeds in the range 5–200 rpm. Tests were conducted for 1 min, and then viscosity values were observed.

### 3.4. Microparticle Preparation

Microparticles were obtained with the spray drying method using a Mini Spray Dryer B-290 (Büchi, Switzerland). To prepare microparticles with POS, the therapeutic substance in appropriate amounts was homogeneously dispersed in the polymer solutions using a magnetic stirrer (Heidolph Instruments, Schabach, Germany) and then spray-dried. After optimization, the drying process parameters were set to: temperatures inlet 150 °C and outlet 86 °C, pressure 80 mm Hg, aspirator blower capacity 85%, and feed rate 2.1 mL/min. Microparticles composition is presented in Table 3.

### 3.5. Moisture Presence

Moisture presence in the obtained microparticles was evaluated by applying moisture analyzer balance (Radwag WSP 50SX, Radom, Poland).

### 3.6. Assessment of Microparticles Morphology

A scanning electron microscope (SEM) with high vacuum mode and secondary electron detector (Inspect^TM^S50, FEI Company, Hillsboro, OR, USA) was applied to evaluate the morphology and shape of prepared ALG and ALG/HPMC microparticles. Before analysis, microparticles were covered with a 6 nm gold layer. SEM analysis was conducted under 10 kV voltage and with 10 mm detector working distance. The microparticles were observed under magnifications of 2000×, 5000×, 10,000×, and 20,000×. All microparticle formulations were also examined with an optical microscope (Motic BA 400, Moticon, Wetzlar, Germany) and observed under 40× magnification.

### 3.7. Estimation of POS Loading, Encapsulation Efficacy, and Production Yield

To evaluate POS loading, 10 mg of microparticles was dissolved in 0.1 M HCl and agitated for 24 h at 75 rpm in a water bath (22 ± 2 °C), successively diluted with methanol, and evaluated spectrophotometrically.

Drug loading (L) was computed using the formula:

L = Q_m_/W_m_ × 100

where Q_m_ is the drug encapsulated in the microparticles and W_m_ is microparticle weight.

The mean drug encapsulation efficiency (EE) was calculated by the expression:

EE = Q_a_/Q_t_ × 100

where Q_a_ is the actual drug content and Q_t_ is the theoretical drug content.

Yield of production (Y) was determined by the formula:

Y = W_m_/W_t_ × 100

where W_m_ is microcapsule weight and W_t_ is the theoretical weight of the drug and polymer.

### 3.8. POS Spectrophotometry Analysis

POS content in the microparticles was evaluated with the spectrophotometry technique using a Jasco Spectrophotometer (Tokyo, Japan). Analysis was performed at a wavelength of 254 nm. The standard calibration curve was linear over the range of 1–50 µg/mL and characterized by the correlation coefficients (R2) 0.9968 (0.1 M HCl) and 0.9997 (SVF). The limit of detection (LOD) and limit of quantification (LOQ) were 0.5 µg/mL and 1.0 µg/mL, respectively.

### 3.9. Swelling Capacity

The swelling capacity was determined using 0.1 M HCl (pH = 1.2) and SVF (pH = 4.2). A total of 20 mg of microparticles was placed in baskets from USP dissolution equipment and situated in Petri dishes with 20 mL of medium. Tests were performed at room temperature (22 ± 2 °C). After specific time intervals, the baskets were taken out, carefully drained, and weighed using analytical balance. The swelling ratio (SR) was calculated by the expression:

SR = (W_S_ − W_0_)/W_0_

where W_0_ is the microparticles’ initial weight and W_S_ is the weight of swollen microparticles. The study was performed in triplicate.

### 3.10. Mucoadhesive Properties

Assessment of mucoadhesive ability was carried out using a TA.XT Plus Texture Analyser (Stable Micro Systems, Godalming, UK). As the mucoadhesive layer, porcine gastric and vaginal mucosa were applied. After moisturizing with 0.1 M HCl or SVF, 10 mg of microparticles was exposed to contact with mucosa with 0.5 N force for 180 s. Experiments were conducted at 22 ± 2 °C. Mucoadhesive properties were expressed as the detachment force (F_max_, recorded by Texture Exponent 32 Software, version 5.0) and the work of mucoadhesion (W_ad_).

### 3.11. In Vitro POS Release

To estimate POS dissolution, a basket apparatus (Erweka Dissolution Tester Type DT 600 HH, Heusenstamm, Germany) was utilized. Microparticles were placed in beakers containing 500 mL 0.1 M HCl (pH = 1.2) or 200 mL SVF (pH = 4.2) and stirred at 100 rpm at 37 ± 1 °C for 24 h. To obtain sink conditions, 1% SDS was added to SVF solution. Samples were taken at the time sections: 5 min, 10 min, 15 min, 20 min, 25 min, 30 min, 45 min, 1 h, 1.5 h, 2 h, 3 h, 4 h, and 5 h, and analyzed spectrophotometrically (Jasco V-750, Tokyo, Japan).

### 3.12. Mathematical Modeling of POS Release Profile

To evaluate the POS release mechanism, results from the POS release test were assessed by using miscellaneous mathematical models:

Zero-order kinetics:

F = k × t

First-order kinetics:

lnF = k × t

Higuchi model:

F = kt^1/2^

Korsmeyer–Peppas model:

F = kt^n^

Hixson–Crowell model:

1 − (1 − F)^1/3^ = kt

where F is the released drug, k is the constant related to the drug release, and t is the time.

### 3.13. Thermal Analysis

Differential scanning calorimetry analysis (DSC) of the unprocessed ALG, HPMC, POS, and spray-dried microparticles was carried out using a Mettler Toledo Star TGA/DSC unit. In DSC analysis, aluminum crucibles with 3–5 mg weighted samples were heated from 0 °C to 300 °C at 20 °C/min under an argon flow; an empty pan was used as the reference.

### 3.14. Antifungal Activity

To evaluate the antifungal activity of the prepared microparticles, the plate diffusion technique was utilized. Fungus inoculum was prepared with sterile 0.9% NaCl solution, reaching the final density 5 × 104 CFU/mL (corresponding to 0.5 on the McFarland scale) [55], and seeded on Petri dishes with a total of 50 µL Sabouraud dextrose agar (SDA). After drying, 10 mg of all tested formulations was immersed in 20 µL of 0.1 M HCl and situated in the middle of the agar plates. POS solution was used as control. The plates with samples were incubated at 37 ± 1 °C for 24 h and 48 h, and after this time the growth inhibition zones were determined by applying a caliper (Mitutoyo, Kawasaki, Japan) with an accuracy of 0.1 mm.

### 3.15. Statistics

Collected data were evaluated by Statistica 13.3 (StatSoft, Tulsa, OK, USA), utilizing one-way analysis of variance (ANOVA) or a Kruskal–Wallis test. The results achieved were implemented as mean and standard deviation on the base of three or six independent experiments. The three-dimensional (3D) response surface was prepared using NCSS 2023 Data Analysis (NCSS LLC, Kaysville, UT, USA) to receive the points of optimum production yield and percent loading.

## 4. Conclusions

ALG and ALG/HPMC combined microparticles were obtained successfully using the spray-drying technique. Both pure ALG and ALG/HPMC microparticles were characterized by mucoadhesive properties, and HPMC improved the mucoadhesiveness of the ALG formulation. A significant impact of HPMC addition on the release profiles was observed; in acidic medium, an accelerated POS release was reported, and in SVF, an extension of release was reported. All ALG and ALG/HPMC formulations demonstrated antifungal activity against *Candida* strains. Taking into account the pharmaceutical characteristics of designed microparticles, it can be concluded that the H5 formulation might be considered as a POS carrier for oral, and the H6 formulation for the vaginal route of POS application. The obtained results indicate that HPMC significantly affects the pharmaceutical properties of ALG microparticles and suggest that they might be utilized to optimize drug delivery in various pH conditions. Further studies aiming to formulate solid oral and vaginal preparations based on designed microparticles are in progress and will be presented in due course.

## Figures and Tables

**Table 1:** Microparticle characteristics (mean ± SD, *n* = 3).

Formulation	Particle Size (µm)	Percent Loading (%)	Encapsulation Efficiency (%)	Production Yield (%)	Moisture Content (%)
A1	10.03 ± 3.84	-	-	63.99 ± 2.93	10.59 ± 1.09 *
A2	10.69 ± 3.49	-	-	63.49 ± 2.77	13.28 ± 0.92
A3	13.42 ± 5.50	-	-	64.47 ± 6.51	14.23 ± 1.53
F1	12.66 ± 4.80	36.21 ± 2.95	118.73 ± 5.44	53.38 ± 4.14	13.12 ± 1.97
F2	13.73 ± 4.63	59.68 ± 2.72 *	108.64 ± 8.84 *	53.23 ± 2.65	8.97 ± 0.49 *
F3	12.81 ± 5.86	37.46 ± 1.41	112.38 ± 4.22	57.72 ± 4.33	11.24 ± 2.22
F4	15.27 ± 5.76	54.30 ± 2.59 *	108.59 ± 5.18 *	56.55 ± 4.11	9.10 ± 1.48 *
F5	11.70 ± 4.81	39.89 ± 1.14	119.67 ± 3.41	53.35 ± 2.95	8.93 ± 1.16 *
F6	13.19 ± 4.01	58.39 ± 2.24 *	116.78 ± 4.48	46.62 ± 3.84 *	10.20 ± 0.81
H1	10.53 ± 3.22	-	-	54.06 ± 5.77	7.54 ± 0.32 *
H2	14.00 ± 4.27	-	-	51.61 ± 4.69	11.87 ± 0.70
H3	15.25 ± 5.12	-	-	52.03 ± 4.58	10.54 ± 2.28
H4	12.23 ± 4.13	37.89 ± 2.39	113.77 ± 7.18	62.46 ± 4.56	9.89 ± 2.18
H5	14.08 ± 3.96	37.41 ± 3.09	112.34 ± 9.28	59.43 ± 1.90	7.60 ± 2.79
H6	15.64 ± 4.48	27.60 ± 2.84 *	138.00 ± 14.21 *	60.05 ± 1.18	13.44 ± 0.35 *

* significant differences (*p* < 0.05).

**Table 2:** Models of POS release from ALG and ALG/HPMC microparticles.

Formulation	Zero-Order Kinetics		First-Order Kinetics		Higuchi Model		Hixson–Crowell Model		Korsmeyer–Peppas Model		
	R^2^	K	R^2^	K	R^2^	K	R^2^	K	R^2^	K	*n*
0.1 M HCl (pH 1.2)											
F1	0.91	2.25	0.98	0.04	0.99	15.42	0.98	113.48	0.99	0.54	0.47
F2	0.88	2.22	0.97	0.04	0.99	17.43	0.98	115.33	0.99	0.55	0.49
F3	0.88	2.15	0.96	0.04	0.99	16.68	0.98	118.49	0.99	0.55	0.48
F4	0.88	2.37	0.98	0.05	0.99	18.31	0.98	107.89	0.99	0.56	0.49
F5	0.90	1.93	0.96	0.03	0.99	13.99	0.99	130.85	0.99	0.54	0.50
F6	0.91	2.72	0.99	0.07	0.99	19.23	0.97	91.96	0.99	0.59	0.53
H4	0.35	1.52	0.47	0.04	0.55	11.32	0.98	104.69	0.75	0.51	0.34
H5	0.24	1.73	0.37	0.03	0.43	13.77	0.95	44.00	0.66	0.54	0.36
H6	0.89	24.06	0.99	1.32	0.93	61.39	0.97	4.66	0.93	0.73	0.57
SVF (pH 4.2)											
F1	0.55	1.63	0.88	0.10	0.69	16.58	0.76	10.84	0.60	0.20	0.14
F2	0.56	1.52	0.99	0.18	0.70	15.51	0.77	8.92	0.58	0.20	0.12
F3	0.62	1.85	0.99	0.15	0.76	18.59	0.89	29.87	0.61	0.18	0.17
F4	0.45	1.42	0.98	0.27	0.60	14.84	0.87	17.76	0.51	0.21	0.12
F5	0.64	1.75	0.86	0.07	0.76	17.60	0.96	60.44	0.64	0.18	0.17
F6	0.88	4.92	0.85	0.43	0.94	34.02	0.86	25.76	0.86	0.22	0.27
H4	0.95	3.37	0.94	0.26	0.99	22.90	0.73	7.06	0.99	0.61	0.51
H5	0.99	5.01	0.89	0.21	0.99	33.64	0.93	49.65	0.99	1.10	1.06
H6	0.92	2.14	0.93	0.11	0.97	20.01	0.97	2.45	0.95	0.82	0.88

**Table 3:** Microparticle composition.

Formulation	ALG Concentration (*w*/*v*%)	HPMC Concentration (*w*/*v*%)	ALG:POS Ratio
A1	0.5	-	-
A2	1	-	-
A3	1.5	-	-
F1	0.5	-	1:0.5
F2	0.5	-	1:1
F3	1	-	1:0.5
F4	1	-	1:1
F5	1.5	-	1:0.5
F6	1.5	-	1:1
H1	1	1	-
H2	1	3	-
H3	1	5	-
H4	1	1	1:1
H5	1	3	1:1
H6	1	5	1:1

## Data Availability

Data are contained within the article; raw data are available upon request.
